# Probing the importance of AIF interaction with endonuclease G in mitochondrial inheritance and neurodegeneration

**DOI:** 10.1038/s41421-024-00736-2

**Published:** 2024-10-22

**Authors:** Shanshan Li, Graham Redweik, Jason L. J. Lin, Yi-Ning Chen, Hanna S. Yuan, Ding Xue

**Affiliations:** 1 School of Life Sciences, Tsinghua University, Beijing, China; 2 Department of Molecular, Cellular and Developmental Biology, University of Colorado, Boulder, CO USA; 3 Institute of Molecular Biology, Academia Sinica, Taipei, Taiwan China

**Keywords:** Cell biology, Developmental biology, Biological techniques

Dear Editor,

Endonuclease G (EndoG) is a conserved nuclease that, under normal conditions, is found in the intermembrane space of mitochondria and plays important roles in multiple cellular processes in diverse organisms, including mitochondrial DNA (mtDNA) replication^1^, apoptosis^2,3^, mitochondrial maintenance^4^, neurodegeneration^5^, and paternal mitochondrial elimination (PME)^6^. Thus, identification and analysis of factors that regulate the activity of EndoG is crucial for understanding how these important cellular events are regulated.

One important EndoG regulator is apoptosis-inducing factor (AIF), a mitochondrial oxidoreductase important for apoptosis^7^. In *C. elegans*, the worm AIF homolog, WAH-1, is found to be an interacting protein and activator of the *C. elegans* EndoG homolog, CPS-6^3,8^. Reduction or loss of *wah-1* activity caused by RNA interference or *wah-1* deletion results in a delay of cell death phenotype similar to that of the *cps-6* mutant^3,8,9^. The activity of CPS-6 is also regulated by oxidation-reduction (redox) conditions^9^. Under reducing conditions, CPS-6 forms a dimeric complex that exhibits high nuclease activity, whereas under oxidative conditions with increased levels of reactive oxygen species, CPS-6 dimers disassociate into monomers with diminished nuclease activity^9^. WAH-1 appears to bind CPS-6 to stabilize the CPS-6 dimer based on in vitro assays. Like AIF, WAH-1 is located in mitochondria and relocated from mitochondria to the nucleus during apoptosis^7,8^, suggesting that WAH-1 and CPS-6 could interact physically in mitochondria and cooperate to affect apoptosis and other cellular processes. In mammals, AIF also plays a role in oxidative phosphorylation and interacts with multiple proteins^10^. Mutations in *AIF* cause neurodegeneration, muscular defects, and various mitochondrial diseases^10,11^. However, loss of AIF or WAH-1 causes embryonic lethality and other severe deficiencies^9,11^, which prevent the characterization of the functions of WAH-1 and AIF in other important cellular processes, especially those regulated by EndoG.

To address these important questions, we attempted to identify residues in WAH-1 that are important for its binding to CPS-6, but do not affect WAH-1’s essential in vivo functions. We constructed a structural model of WAH-1 monomer using the crystal structure of human AIF (PDB entry: 1M6I) as the template by 3D-JIGSAW. We then performed docking simulations between WAH-1 monomer and CPS-6 dimer (PDB entry: 3S5B) using ZDOCK. In the constructed structural model of the WAH-1 and CPS-6 complex (Fig. 1a), Arg 473 of WAH-1 locates at the center of the interface, suggesting that this residue could be important for the WAH-1 and CPS-6 interaction. In an in vitro binding assay, the nuclease-defective CPS-6(H148A) protein showed a dissociation constant (*K*d) of 84.5 nM in binding to WAH-1^9^, as determined by intrinsic tryptophan fluorescence (Supplementary Fig. S1a), whereas WAH-1(R473A) and WAH-1(R473E) proteins respectively containing the R473A and the R473E substitutions showed 1.4-fold and 4.5-fold higher *K*d in association with CPS-6(H148A), respectively (Supplementary Fig. S1a), indicating that R473 is an important interface residue for WAH-1 and CPS-6 interaction.

**Fig. 1:**
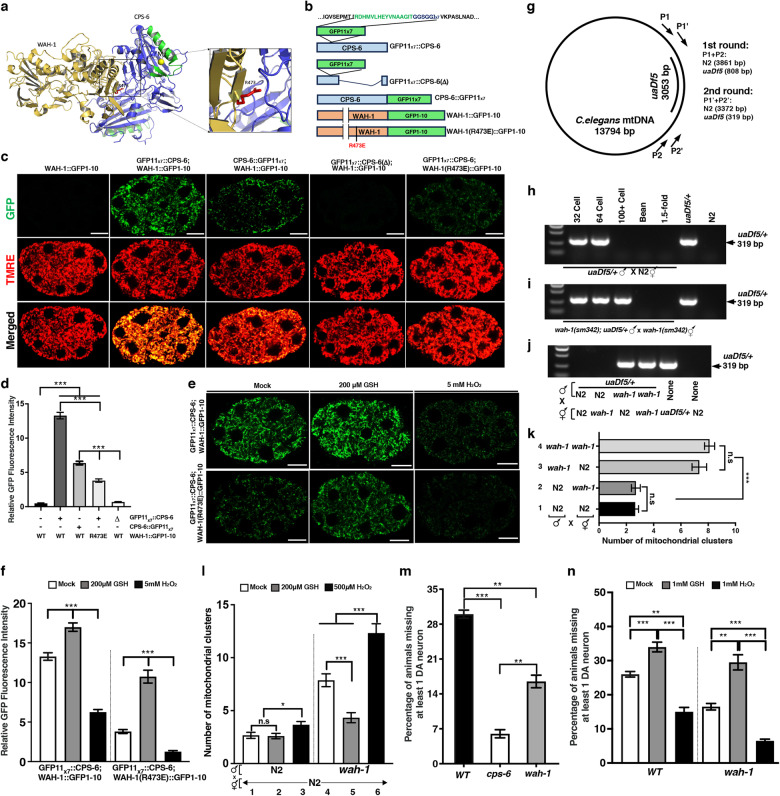
WAH-1 interacts with CPS-6 in mitochondria and promotes PME and α-synuclein-induced DA neuronal death*.* **a** A CPS-6/WAH-1 structural model generated via ZDOCK, in which a CPS-6 dimer (blue) interacts with one WAH-1 monomer (brown), with the Mg^2+^ ion (yellow) interacting with the catalytic His-Me finger motif (green) in CPS-6. Arg473 (red) in WAH-1, shown in an enlarged view, is identified as a key interfacial residue between WAH-1 and CPS-6. **b** A schematic diagram of the GFP11_x7_ tag and GFP1–10 tag inserted into the *cps-6* and *wah-1* loci, respectively, by genome editing. The sequence of GFP11 is shown. In the GFP11_x7_::CPS-6(Δ) strain, CPS-6 residues 114–295 are deleted. **c**, **e** Representative GFP, TMRE and merged images of embryos (**c**) or GFP images of embryos (**e**) carrying the indicated knockins at the stage of 6 to 8 cells without (**c**) or with the indicated treatments (**e**). The mock treatment was M9 buffer. Scale bars, 10 µm. **d**, **f** Quantification of relative GFP fluorescence intensity of embryos from the indicated strains with or without the indicated treatment, *n* ≥ 25. Data are mean ± SEM. **g** A diagram of *C. elegans* mtDNA, the *uaDf5* deletion, primers and expected sizes of PCR products in each round of the nested PCR assays. **h**–**j** Representative gel images of PCR products from 15 cross-fertilized embryos (MTR-positive) at the indicated stages (**h**, **i**) or 100-cell stage (**j**) from the indicated crosses with MTR-stained males. PCR controls are N2 or *uaDf5/+* embryos. **k**, **l** Quantification of MTR-stained paternal mitochondrial clusters in 64-cell embryos from the indicated crosses with MTR-stained males without or with the indicated treatment. Data are mean ± SEM; *n* = 20 (**k**) or 15 (**l**) per cross. **m**, **n** Comparison of DA neuronal loss in adult day 2 *baIn11* animals in the indicated genetic background without or with the indicated treatment. Data are mean ± SEM, *n* = 50 animals per experiment in 4 independent experiments for each genotype and condition. *P < 0.05; **P < 0.01; ***P < 0.001; ns not significant (one-way ANOVA; **d**, **f**, **k**–**n**). *wah-1(sm342*[R437E]*)* and *cps-6(tm3222)* alleles were used.

Using the CRISPR/Cas9 genome editing method^12^, we introduced the R473E substitution into the *wah-1* gene in *C. elegans*. The resulting mutant, *wah-1(sm342*[R473E]*)*, exhibited a comparable growth rate, embryonic lethality rate, and brood size to those of wild-type (WT) N2 animals and the *cps-6(tm3222)* loss-of-function mutant (Supplementary Fig. S1b–d), indicating that the R473E mutation has a minimal impact on *C. elegans* development and could assist the analysis of WAH-1 in vivo functions.

WAH-1 has been shown to bind CPS-6 in vitro to enhance the endonuclease activity of CPS-6^8,9^. However, whether this protein interaction occurs in vivo and is important for functions of both proteins is unclear. To address this important question, we employed the Bimolecular Fluorescence Complementation (BiFC) assay, using the split GFP system with GFP1–10 and the tandem GFP11 tag (GFP11_x7_) to enhance the fluorescence intensity (Fig. 1b)^13^. Using CRISPR/Cas9 genome editing, we inserted the GFP11_x7_-coding sequence into the *cps-6* locus, creating an in-frame insertion near the N-terminus of CPS-6, right after the mitochondrial targeting sequence (GFP11_x7_::CPS-6, Fig. 1b). We also generated a GFP11_x7_ knockin at the C-terminus of CPS-6 to produce CPS-6::GFP11_x7_. Conversely, GFP1–10 knockin at the C-terminus of WAH-1 in the *wah-1* locus and in the *wah-1* locus harboring the R473E mutation was generated to produce WAH-1::GFP1–10 and WAH-1(R473E)::GFP1–10 (Fig. 1b), respectively. Because GFP11_x7_::CPS-6 and WAH-1::GFP1–10 knockin animals exhibited a comparable growth rate, embryonic lethality rate, and brood size to those of N2 animals (Supplementary Fig. S1b–d), the GFP tags do not appear to affect the physiological functions of CPS-6 and WAH-1.

As expected, embryos from WAH-1::GFP1–10 animals did not have any fluorescence (Fig. 1c, d), due to the absence of GFP11 required to restore GFP fluorescence. Interestingly, animals carrying both WAH-1::GFP1–10 and GFP11_x7_::CPS-6 knockins yielded strong GFP signals in the cytoplasm that were excluded from the nuclei, while animals carrying WAH-1::GFP1–10 and CPS-6::GFP11_x7_ knockins exhibited significantly weaker GFP signals with the same pattern (Fig. 1c, d), suggesting that WAH-1::GFP1–10 restores GFP fluorescence with either GFP11_x7_::CPS-6 or CPS-6::GFP11_x7_, but the position of GFP11_x7_ in CPS-6 affects the efficiency of GFP reconstitution. We then generated an in-frame deletion in the *cps-6* coding region in GFP11_x7_::CPS-6 knockin animals (Fig. 1b), removing most of the CPS-6 protein (residues 114–295). The resulting knockin, GFP11_x7_::CPS-6(Δ), failed to reconstitute GFP with WAH-1::GFP1–10 (Fig. 1c, d), suggesting that CPS-6 and WAH-1 interaction is crucial for restoring GFP in animals with WAH-1::GFP1–10 and GFP11_x7_::CPS-6 knockins. Importantly, animals carrying WAH-1(R473E)::GFP1–10 and GFP11_x7_::CPS-6 knockins showed significantly reduced GFP fluorescent intensity (Fig. 1c, d), but comparable WAH-1 expression levels to animals carrying WAH-1::GFP1–10 and GFP11_x7_::CPS-6 knockins (Supplementary Fig. S1e). These results suggest that the R473E mutation impairs the interaction between WAH-1 and CPS-6 and thus the intensity of the reconstituted GFP. Together, these results demonstrate for the first time that CPS-6 and WAH-1 interact in vivo.

Both WAH-1 and CPS-6 localize to mitochondria^3,8^. The reconstituted GFP in animals carrying WAH-1::GFP1–10 and GFP11_x7_::CPS-6 or CPS-6::GFP11_x7_ completely colocalized with tetramethylrhodamine ethyl ester (TMRE) (Fig. 1c; Supplementary Fig. S2), a mitochondrion-specific fluorescent dye^6^. Although the WAH-1(R473E) mutation impaired binding between WAH-1 and CPS-6, it did not affect colocalization of the weak, reconstituted GFP with TMRE in mitochondria (Fig. 1c; Supplementary Fig. S2). These results suggest that CPS-6 and WAH-1 interact and colocalize exclusively in mitochondria.

Oxidative stress has been shown to decrease the nuclease activity of CPS-6 by impairing CPS-6 dimer formation and CPS-6 interaction with WAH-1 in vitro^9^. We found that treatment of animals carrying WAH-1::GFP1-10 and GFP11_x7_::CPS-6 with an antioxidant, reduced glutathione (GSH), significantly enhanced the fluorescent intensity of reconstituted GFP (Fig. 1e, f). By contrast, treatment of the same animals with an oxidant, H_2_O_2_, significantly reduced the fluorescent intensity of reconstituted GFP (Fig. 1e, f). In comparison, GSH or H_2_O_2_ treatment of WAH-1::GFP knockin animals did not alter WAH-1::GFP fluorescent intensity (Supplementary Fig. S3a, b). These results indicate that GSH treatment enhances and H_2_O_2_ treatment decreases WAH-1 and CPS-6 interaction in vivo. Moreover, GSH treatment markedly enhanced and H_2_O_2_ treatment nearly abolished GFP fluorescence in animals carrying WAH-1(R473E)::GFP1–10 and GFP11_x7_::CPS-6 (Fig. 1e, f), indicating that antioxidant treatment rescues and oxidant treatment exacerbates impaired WAH-1 and CPS-6 in vivo interaction caused by WAH-1(R473E). Taken together, these results indicate that the redox conditions can have important, regulatory effects on WAH-1 and CPS-6 in vivo interaction, and potentially, on their in vivo functions. The finding that the intensities of reconstituted GFP in animals carrying WAH-1::GFP1-10 and GFP11_x7_::CPS-6 correlate with the redox conditions suggests that the WAH-1::GFP1-10/GFP11_x7_::CPS-6 pair can be used as an in vivo redox sensor.

Because CPS-6 acts in paternal mitochondria to facilitate PME through degrading mtDNA^6^, we tested whether WAH-1 and its interaction with CPS-6 are important for PME. Using a 3053-bp mtDNA deletion allele (*uaDf5*) and a sensitive PCR-based method to track paternal mtDNA (Fig. 1g), we could detect *uaDf5* paternal mtDNA in cross-fertilized embryos at or before 64-cell stage from mating of *uaDf5/+* heteroplasmic males with N2 hermaphrodites, but not in later embryonic stages (Fig. 1h)^6^. In mating between *wah-1(sm342*[R473E]*); uaDf5/+* males with *wah-1(sm342*[R473E]*)* hermaphrodites, *uaDf5* paternal mtDNA was detected in embryos around 100-cell stage (Fig. 1i), indicating that PME is delayed by the WAH-1(R473E) mutation and *wah-1* is important for PME. We investigated the paternal or maternal requirement for *wah-1* in PME and found that delayed removal of *uaDf5* paternal mtDNA was only observed when male parents carried the *wah-1(sm342*[R473E]*)* mutation regardless of the maternal genotype (Fig. 1j), indicating that the paternal but not maternal *wah-1* activity is required for PME. This paternal requirement for *wah-1* in PME is identical to that of CPS-6^6^, with which WAH-1 interacts to activate. The PME delay phenotype caused by *wah-1(sm342*[R473E]*)* is weaker than that caused by *cps-6(tm3222)*^6^, probably because the WAH-1(R473E) mutation decreases, but does not abolish the binding between WAH-1 and CPS-6.

We confirmed these results using a different PME assay, the fluorescence microscopy analysis, which monitors the disappearance of paternal mitochondria stained by Mitotracker Red (MTR)^6^, another mitochondrion-specific dye. When MTR-stained males were mated with unstained hermaphrodites, significantly more MTR-stained paternal mitochondrial clusters were seen in 64-cell cross-fertilized embryos from *wah-1(sm342*[R473E]*)* male parents than those from N2 male parents regardless of the maternal genotype (Fig. 1k; Supplementary Fig. S4a, b), indicating the paternal requirement for *wah-1* in PME. Since a revertant allele of *wah-1(sm342*[R473E]*)* created by genome editing, *wah-1(sm342 sm1038*[E473R]*)*, did not cause any PME defect (Supplementary information, Fig. S3c, d), the *wah-1(sm342*[R473E]*)* mutation is solely responsible for the PME defect. These results confirm that WAH-1, like CPS-6, acts paternally to promote PME.

We next examined whether redox conditions affect PME through regulating CPS-6 and WAH-1 interaction. GSH treatment did not obviously affect PME in WT embryos and did not reduce the already low number of paternal mitochondrial clusters observed in 64-cell stage embryos from mating of MTR-stained N2 males with N2 hermaphrodites (Fig. 1l; Supplementary Fig. S4c, d). In comparison, H_2_O_2_ treatment slightly increased the number of paternal mitochondrial clusters in 64-cell stage embryos from the same cross, indicating that H_2_O_2_ oxidant could compromise PME in WT embryos, which is consistent with the finding that H_2_O_2_ treatment reduced WAH-1/CPS-6 binding (Fig. 1e, f). Importantly, treatment of MTR-stained *wah-1(sm342*[R473E]*)* males with GSH or H_2_O_2_ significantly reduced or enhanced the PME defect observed in cross-fertilized embryos with unstained N2 hermaphrodites (Fig. 1l; Supplementary Fig. S4c, d), respectively, confirming that enhancing WAH-1(R473E)/CPS-6 binding by GSH and reducing WAH-1(R473E)/CPS-6 binding by H_2_O_2_ increase and decrease PME in vivo, respectively, and that the WAH-1 and CPS-6 interaction is crucial for PME.

Following fertilization, paternal mitochondria are depolarized and undergo internal breakdown, leading to relocation of CPS-6 from the intermembrane space of paternal mitochondria to the mitochondrial matrix to degrade mtDNA^6^, which facilitates self-destruction of paternal mitochondria. We examined whether WAH-1 and CPS-6 maintain interaction following fertilization. MTR-stained N2 males or GFP11_x7_::CPS-6; WAH-1::GFP1–10 males were mated with *fog-2(q71)* females, which cannot produce sperm and self-fertilize, but could mate with males. Before fertilization, the reconstituted GFP fluorescence from GFP11_x7_::CPS-6 and WAH-1::GFP1–10 was completely colocalized with MTR-stained sperm mitochondria (Supplementary Fig. S5a). After fertilization, the reconstituted GFP fluorescence from WAH-1::GFP1–10/GFP11_x7_::CPS-6 remained visible and fully colocalized with MTR-stained paternal mitochondria (Supplementary Fig. S5b, c), indicating that WAH-1 maintains binding to CPS-6 in paternal mitochondria after fertilization and relocates with CPS-6 to paternal mitochondrial matrix. This is consistent with WAH-1 acting paternally with CPS-6 to promote PME.

Given the role of EndoG in mediating dopaminergic neuronal death caused by α-synuclein expression in multiple species^5^, we examined whether the WAH-1 and CPS-6 interaction is important for this neurodegeneration process. In a *C. elegans* Parkinson’s disease model, which carries an integrated transgene (*baIn11*) co-expressing GFP and human α-synuclein in dopaminergic (DA) neurons (P*dat-1::gfp*/P*dat-1*::α-synuclein)^14^, 30% of adult day 2 animals lost at least one DA neuron (Fig. 1m). This DA neuronal death was strongly suppressed by the *cps-6*(*tm3222*) deletion mutation (Fig. 1m), confirming that CPS-6 is required for α-synuclein-induced neuronal death. Interestingly, the *wah-1(sm342*[R473E]*)* mutation (Fig. 1m), but not the *wah-1(sm342 sm1038*[E473R]*)* revertant (Supplementary Fig. S3e), resulted in 50% reduction in DA neuronal death, suggesting that the WAH-1 and CPS-6 interaction is important for mediating α-synuclein-induced neurodegeneration.

Moreover, treatment with the antioxidant, GSH, increased neurodegeneration in *baIn11* animals and restored DA neuronal death in *wah-1(sm342*[R473E]*); baIn11* animals to the level seen in *baIn11* animals, compared to mock treatments in respective backgrounds (Fig. 1n). Conversely, treatment with the oxidant, H_2_O_2_, significantly reduced neurodegeneration in *baIn11* animals and nearly blocked DA neuronal death in *wah-1(sm342*[R473E]*); baIn11* animals (Fig. 1n). These results are consistent with the findings that GSH treatment enhanced and H_2_O_2_ treatment reduced the interaction of CPS-6 with WAH-1 and WAH-1(R473E) (Fig. 1e, f). Taken together, these results demonstrate that WAH-1 and its interaction with CPS-6 are crucial for α-synuclein-induced DA neurodegeneration.

The split GFP technology has been used to investigate protein–protein interactions, protein localization, cell–cell contacts, and other important cellular events^13,15^. In most studies, one or both of the split GFP fusions with target proteins are overexpressed, which could enhance reconstituted GFP signals, but may lead to artificial or exaggerated interactions of fusion proteins^13,15^. Visualization of the interaction between endogenous proteins using the GFP1–10 and GFP11 split GFP system has not been reported before. In this study, we report successful visualization of protein interaction between two endogenous *C. elegans* mitochondrial proteins, CPS-6 and WAH-1, using the split GFP assay. This in vivo protein interaction assay aids in the identification of a viable mutation (R473E) in the *wah-1* gene that impairs protein interaction between WAH-1 and CPS-6 and allows analysis of the physiological consequences of this protein interaction, leading to the discovery of two previously unknown functions of WAH-1 in mediating neurodegeneration and PME. Moreover, this in vivo protein interaction is reduced and enhanced by oxidation and reduction conditions, respectively, leading to a corresponding decrease and increase of the WAH-1 functions in *C. elegans*. This correlative demonstration of in situ endogenous protein interactions and protein functions, and under different cellular environments such as different redox conditions, have not been reported before and will have broad and important applications, including screens to identify compounds that alter a specific protein interaction in related human diseases, such as Parkinson’s disease and cancer.

## Supplementary information

Probing the importance of AIF interaction with endonuclease G in mitochondrial inheritance and neurodegeneration
