# Supplementary material for: Probing the importance of AIF interaction with endonuclease G in mitochondrial inheritance and neurodegeneration
Source: Cell Discov. 2024 Oct 22;10:107. doi: 10.1038/s41421-024-00736-2 (PMC11496498; doi:10.1038/s41421-024-00736-2)
Supplement: Supplementary file 1 — Probing the importance of AIF interaction with endonuclease G in mitochondrial inheritance and neurodegeneration [file 41421_2024_736_MOESM1_ESM.pdf]

Supplementary information

Materials and methods

***C. elegans* Strains**

C. elegans strains were maintained at 20°C on nematode growth medium (NGM) plates seeded with *E. coli* OP50 bacteria using standard methods.[1] The N2 Bristol strain was used as the wild-type strain. The alleles used in this study: LGI, *cps-6(tm3222), cps-6(sm822[gfp11$_{x7}$::cps-6]), cps-6(sm1033[cps-6::gfp11$_{x7}$]), cps-6(sm995[gfp11$_{x7}$::cps-6(Δ)])*; LGIII, *wah-1(sm321[wah-1::gfp])*[2], *wah-1(sm342[R473E]), wah-1(sm984[wah-1::gfp1-10]), wah-1(sm1035[R473E]sm984[wah-1::gfp1-10]),* LGV, *fog-2(q71)*[3]; mtDNA, *uaDf5*. *baIn11* is an integrated transgene co-expressing GFP and human α-synuclein in dopaminergic (DA) neurons under the control of the *dat-1* gene promoter (P*dat-1::gfp*/P*dat-1*::α-synuclein).[4]

Measurement of the binding affinity between CPS-6 and WAH-1

The His-tagged CPS-6(H148A) proteins (residues 21-308) and the WAH-1 proteins (residues 214-700) were expressed and purified according to the previously described protocol.[5] Constructs expressing two WAH-1 mutants, WAH-1(R473A) and WAH-1(R473E), were generated by Quickchange site-directed mutagenesis kits (Stratagene). Both WAH-1 mutants were purified using the same protocol as that of wild-type WAH-1. The intrinsic tryptophan fluorescence signals were measured to monitor the interactions between CPS-6 and WAH-1 proteins using a Varian Cary Eclipse Fluorescence Spectrophotometer (Agilent Technologies) at 25ºC. Fluorescence spectra were obtained using an excitation wavelength of 296 nm and emission scans were performed at 0.5 nm intervals between 300 to 500 nm. The CPS-6 and WAH-1 binding curves were calculated by subtraction of the intrinsic tryptophan fluorescence signals of CPS-6 alone from the ones after the addition of CPS-6 at a concentration range of 0 to 0.65 μM to WAH-1 (65 nM) at λmax = 341 nm. The CPS-6 and WAH-1 dissociation constant (Kd) value was calculated using the non-linear regression program GraphPad Prism 4 (GraphPad Inc.). Wild-type CPS-6 proteins are highly toxic to *E. coli*. The H148A mutation in the active site of CPS-6 does not change the protein overall conformation, its DNA-binding ability, and its interaction with WAH-1.[5,6] CPS-6(H148A) was thus expressed, purified and used in the WAH-1 binding assays.

Structural modeling of the CPS-6/WAH-1 complex

The structural model of the WAH-1 monomer was built using the crystal structure of human AIF (PDB entry: 1M6I) as the template by 3D-JIGSAW.[7] The complex structural model of WAH-1 monomer and CPS-6 dimer (PDB entry: 3S5B) was constructed using ZDOCK.[8]

Growth rate assays

In this experiment, 30 gravid adults were individually placed on separate NGM plates to lay eggs for 30 minutes before they were removed. After 9 hours, the time that each L1 larva reached the early L4 stage was recorded and used to evaluate the growth rate.

Embryonic lethality assays

The assays were carried out at 25°C. Late L4 larvae were aged for 12 hours before being transferred to separate fresh plates to lay eggs for 6 hours. Subsequently, the total number of eggs laid by each animal on the plates was counted. After 24 hours, embryos that failed to hatch were scored as dead embryos. The embryonic lethality rate was determined by dividing the number of dead eggs by the total number of eggs laid.

Brood size assays

Thirty late L4 larvae were individually placed on separate OP50-seeded NGM plates. Every other day, the animals were transferred to new NGM plates until they reached the end of their lifespans. The number of eggs and larvae on each plate was recorded, and the total number of eggs and larvae produced by each animal was used to determine the brood size of the animal.

Generation of knock-in strains

Generation of knockin mutations or insertions by CRISPR/Cas9 genome editing was performed as described previously.[9,10] The sgRNA sequences and the repair template sequence used in each knockin experiment are shown in Table S1 and Fig. S6, respectively. Primers used to screen for the knockin mutation or insertion are shown in Table S2.

Fluorescent microscopy and TMRE staining of embryos

Tetramethyl rhodamine methyl ester (TMRE, Invitrogen, Cat: T669) dissolved in DMSO (10 mM) was diluted to a final concentration of 6 μM and then applied to the OP50 bacterial lawn on NGM plates (TMRE-NGM). L4 larval stage hermaphrodites were placed in the TMRE-NGM plates and incubated overnight in dark at 20°C. After that, they were moved to new NGM plates with fresh OP50 bacterial lawn to remove TMRE from their exteriors, before they were dissected to release embryos onto an agarose pad. A coverslip was gently placed over the embryos, and M9 buffer was pipetted into the space between the coverslip and the slide. A hot mixture of petrol gel and beeswax was then used to seal the sample. Visualization of the embryos was performed using excitation lasers at 488 nm for GFP and 561 nm for TMRE, utilizing a Zeiss microscope equipped with a vertical-stage laser scanning confocal system (Zeiss LSM900). Exposure time, laser strength, and other settings were identical in acquiring images of all embryos.

Quantification of GFP fluorescence intensity

The GFP fluorescence intensity was quantified from deconvolved images of embryos. Embryos were obtained from dissected adult hermaphrodites and images of embryos were captured using Zeiss LSM900 confocal microscope and analyzed using the Image J software.

MitoTracker Red (MTR) staining of males

1 mM stock solution of MitoTracker Red (MTR) (Invitrogen Cat. M7512) dissolved in dimethyl sulfoxide (DMSO) was diluted in the M9 buffer (42.3 mM Na_2HPO4, 22 mM KH_2PO_4, 8.6 mM NaCl, 18.7 mM NH_4Cl) to a final concentration of 50 μM before mixing with the OP50 bacteria. L4 larval stage males were exposed to the MTR/bacteria mixture seeded on NGM plates for 12 hours in dark. Subsequently, they were transferred to fresh NGM plates seeded with OP50 bacteria three times for 15 minutes each to undergo three rounds of "bacterial showers" to remove MTR from their exteriors. After that, MTR-stained males were mated with unstained young adult hermaphrodites or *fog-2(q71)* females for 8 hours. The mated hermaphrodites or females were dissected to obtain MTR-stained, cross-fertilized embryos. To obtain MTR-stained sperms, MTR-

stained males were dissected to release sperms from gonads onto an agarose pad with M9 buffer for imaging analysis. Images of embryos or sperms were captured for GFP (488 nm) and MTR-stained mitochondria (561 nm) via the Zeiss LSM900 confocal microscope.

PCR detection of the *uaDf5* mtDNA deletion

Hermaphrodites mated with MTR-stained *uaDf5*/+ males were selected based on their spermathecae filled with MTR-stained sperms and dissected to obtain early cross-fertilized embryos or used to collect later stage embryos. Multiple cross-fertilized embryos at the same stage were pooled and subjected to two rounds of nested PCR analysis to detect the *uaDf5* mtDNA deletion. For the first round of PCR, primers P1 (5'-GATTAGCACAAGCTTTATTGGATGG-3') and P2 (5'-AAGATCTTAACATTCCGGCTGAGGC-3') near the *uaDf5* deletion region were used. For the second round of PCR, primers P3 (5'-CCATCCGTGCTAGAAGACAA-3') and P4 (5'-CTTCTACAGTGCATTGACCTAGTC-3') were used.

Confocal microscopy and quantification of MTR-stained paternal mitochondrial clusters

Z-stack images were captured from the top to the bottom of the embryo with a 2 μm distance between stacks using a Zeiss LSM 900 microscope. Subsequently, fluorescent images were deconvolved and projected into a single plane. The number of paternal mitochondrial clusters labeled by MTR was quantified from the processed images of embryos.

Treatment of animals with reduced glutathione (GSH) or H_2O_2.

GSH and H_2O_2 were initially dissolved in the M9 buffer at concentrations of 1 M or 10 mM, respectively. GSH solutions were then diluted into liquid NGM to a final concentration of 200 μM before making NGM plates. For GSH treatments, L4 stage hermaphrodites were grown on NGM plates containing 200 μM GSH and their adult hermaphrodite progeny were dissected to obtain embryos for imaging analysis. For PME assays, males were grown on NGM plates containing 200 μM GSH, starting from L1 larvae. L4 males were then stained by MTR on fresh GSH NGM plates before they were mated with N2 hermaphrodites on GSH NGM plates for 8 hours. Their F1 embryos were then quantified for MTR-stained paternal mitochondrial clusters. For H_2O_2

treatments, adult hermaphrodites were soaked in 5mM H_2O_2 solutions for 1 hour before they were subjected to imaging analysis. For PME assays, MTR-stained L4 stage males were soaked in 500 μM H_2O_2 for 1 hour, let recover on NGM plates for 1 hour, and soaked in 500 μM H_2O_2 for one additional hour, before they were mated with unstained N2 hermaphrodites. For mock treatments, animals were treated with the M9 buffer only.

Quantification of dopaminergic neuronal death.

L4 stage *baIn11* animals (P*dat-1::gfp*/P*dat-1*::α-synuclein) carrying the *cps-6(tm3222)* or *wah-1(sm342*[R473E]*)* alleles were maintained on OP50-seeded NGM plates or subjected to treatments with either M9 buffer (Mock), GSH (1 mM), or H_2O_2 (1 mM). The quantification of dopaminergic (DA) neuronal death was performed on adult day 2 animals, where the percentage of animals exhibiting the loss of at least one out of six DA neurons (labeled with GFP) in the head was scored via Nomarski fluorescent microscope. Experiments were repeated four times, with 50 animals being scored per experiment.

Enhanced slowing response (ESR) locomotion assay

The ESR assay was performed as described previously.[11] Adult day 2 animals were washed two times with the M9 buffer and then incubated with the M9 buffer for 1 hour under rotation at room temperature. Thereafter, animals were washed again with the M9 buffer and transferred to empty or OP50-seeded NGM plates (20-30 animals per plate). Animals were allowed to recover on plates for 5 minutes before their movements were tracked for 1 minute via the WormLab system (MBF Bioscience). Body bends of each animal per minute were then recorded. To calculate slowing response rate, the average numbers of body bends on OP50-seeded NGM plates (BB_F) and on empty NGM plates (BB_E) were used in the following equation:

Slowing response rate = $[(BB_F - BB_E)/BB_E]$ x100.

We used this (ESR) locomotion assay to examine if loss of DA neurons in *baIn11* animals is associated with hyperactive locomotion on foods and found that the *wah-1(sm342*[R473E]*)* mutation and the *cps-6(tm3222)* mutation partially blocked and strongly suppressed such hyperactivity seen in *baIn11* animals, respectively (Supplementary Fig. S3f).

Immunoblotting analysis

Animals at the L4 stage were placed in the SDS sample buffer and boiled for 20 minutes. The samples were resolved by 10% SDS polyacrylamide gel and transferred to a PVDF membrane. WAH-1 GFP fusion proteins and Tubulin were detected using a monoclonal anti-GFP antibody (Abmart, M20004S, 1:1000 dilution), a monoclonal anti-Tublin antibody (Bioss, bsm-8772M, 1:1000 dilution), and a goat-anti-mouse secondary antibody conjugated with horseradish peroxidase (Cell Signaling, 7076S, 1:10000 dilution).

Statistical analysis

One-way ANOVA test was utilized to evaluate the significance of the difference between different groups of data. Statistical analyses were conducted using the GraphPad Prism 9.0 software (GraphPad Software, Inc., San Diego, CA).

1. Brenner, S. The genetics of Caenorhabditis elegans. *Genetics* **77**, 71–94 (1974).
2. Wang, Y. *et al*. Kinetics and specificity of paternal mitochondrial elimination in Caenorhabditis elegans. *Nature Communications* **7**, DOI: 10.1038/ncomms12569 (2016).
3. Clifford, R. *et al*. FOG-2, a novel F-box containing protein, associates with the GLD-1 RNA binding protein and directs male sex determination in the C. elegans hermaphrodite germline. *Development* **127**, 5265–76 (2000).
4. Kautu, B. B., Carrasquilla, A., Hicks, M. L., Caldwell, K. A. & Caldwell, G. A. Valproic Acid Ameliorates C. elegans Dopaminergic Neurodegeneration with Implications for ERK-MAPK Signaling. *Neurosci Lett* **541**, 116–119 (2013).

5. Lin, J. L. J. *et al*. Oxidative Stress Impairs Cell Death by Repressing the Nuclease Activity of Mitochondrial Endonuclease G. *Cell Rep* **16**, 279–287 (2016).

6. Lin, J. L. *et al*. Structural insights into apoptotic DNA degradation by CED-3 protease suppressor-6 (CPS-6) from Caenorhabditis elegans. *J Biol Chem* **287**, 7110–20 (2012).

7. Bates, P. A., Kelley, L. A., MacCallum, R. M. & Sternberg, M. J. Enhancement of protein modeling by human intervention in applying the automatic programs 3D-JIGSAW and 3D-PSSM. *Proteins* **Suppl 5**, 39–46 (2001).

8. Pierce, B. G. *et al*. ZDOCK server: interactive docking prediction of protein–protein complexes and symmetric multimers. *Bioinformatics* **30**, 1771–1773 (2014).

9. Zhao, P. *et al*. One-step homozygosity in precise gene editing by an improved CRISPR/Cas9 system. *Cell Res* **26**, 633–6 (2016).

10. Zhao, P., Zhang, Z., Ke, H., Yue, Y. & Xue, D. Oligonucleotide-based targeted gene editing in C. elegans via the CRISPR/Cas9 system. *Cell research* **24**, 247–50 (2014).

11. Petratou, D., Fragkiadaki, P., Lionaki, E. & Tavernarakis, N. Assessing locomotory rate in response to food for the identification of neuronal and muscular defects in *C. elegans*. *STAR Protocols* **5**, 102801 (2024).

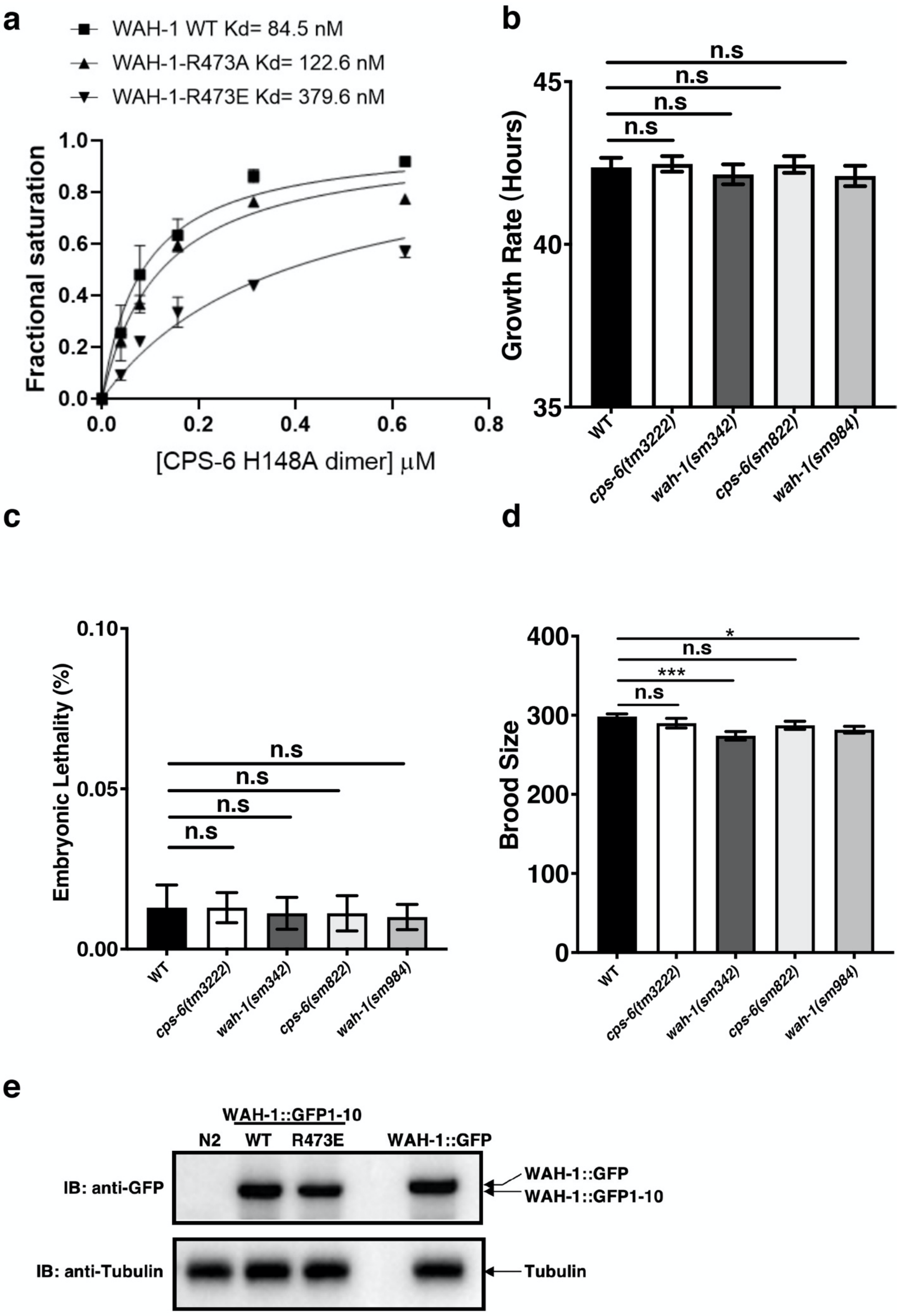

a
WAH-1 WT Kd= 84.5 nM
WAH-1-R473A Kd= 122.6 nM
WAH-1-R473E Kd= 379.6 nM
Fractional saturation
1.0
0.8
0.6
0.4
0.2
0.0
0.0
0.2
0.4
0.6
0.8
[CPS-6 H148A dimer] μM
b
n.s
n.s
n.s
n.s
Growth Rate (Hours)
45
40
35
WT
cps-6(tm3222)
wah-1(sm342)
cps-6(sm822)
wah-1(sm984)
c
Embryonic Lethality (%)
0.10
0.05
0.00
n.s
n.s
n.s
n.s
WT
cps-6(tm3222)
wah-1(sm342)
cps-6(sm822)
wah-1(sm984)
d
Brood Size
400
300
200
100
0
*
n.s
***
n.s
WT
cps-6(tm3222)
wah-1(sm342)
cps-6(sm822)
wah-1(sm984)
e
WAH-1::GFP1-10
N2
WT
R473E
WAH-1::GFP
IB: anti-GFP
WAH-1::GFP
WAH-1::GFP1-10
IB: anti-Tubulin
Tubulin

Fig. S1. The Arg473 residue in WAH-1 is a key interface residue in the CPS-6/WAH-1 complex and its substitution by Glu has minimal impact on *C. elegans* development.

a The dissociation constant (Kd) between CPS-6 and WAH-1 (wild-type, R473A, and R473E) was determined by intrinsic tryptophan fluorescence. Purified nuclease-defective CPS-6(H148A) protein and $Histine_6$-tagged WAH-1(214–700) proteins (wild-type, R473A, and R473E) were used in the binding assays. The interaction between CPS-6 and WAH-1 proteins increased fluorescence emission at the wavelength of 341 nm (by an excitation wavelength of 296 nm), which was detected in the intrinsic tryptophan fluorescence spectra. **b-d** The growth rate (**b**), the embryonic lethality rate (**c**), and the brood size (**d**) of wild-type (WT), *cps-6(tm3222)*, *wah-1(sm342[R473E])*, *cps-6(sm822[gfp11*$_{x7}$*::cps-6])*, and *wah-1(sm984[wah-1::gfp1-10])* animals are shown. n = 30 (**b**, **d**) or 17 (**c**) for each experiment. Data are mean ± SEM. ****P* < 0.001, **P* < 0.05 (One-way ANOVA test); n.s, not significant. **e** Immunoblotting analysis of N2, *cps-6(sm822[gfp11*$_{x7}$*::cps-6]); wah-1(sm984[wah-1::gfp1-10]), cps-6(sm822[gfp11*$_{x7}$*::cps-6]); wah-1(sm1035[R473E] sm984[wah-1::gfp1-10]),* and *wah-1(sm321[wah-1::gfp])* animals. Equal amounts of lysates from the indicated L4 stage animals were resolved by 10% SDS polyacrylamide gel. Blots were probed with an anti-GFP and an anti-Tubulin mouse monoclonal antibody, respectively, and detected using HRP-conjugated goat anti-mouse secondary antibody. Tubulin was used as a loading control.

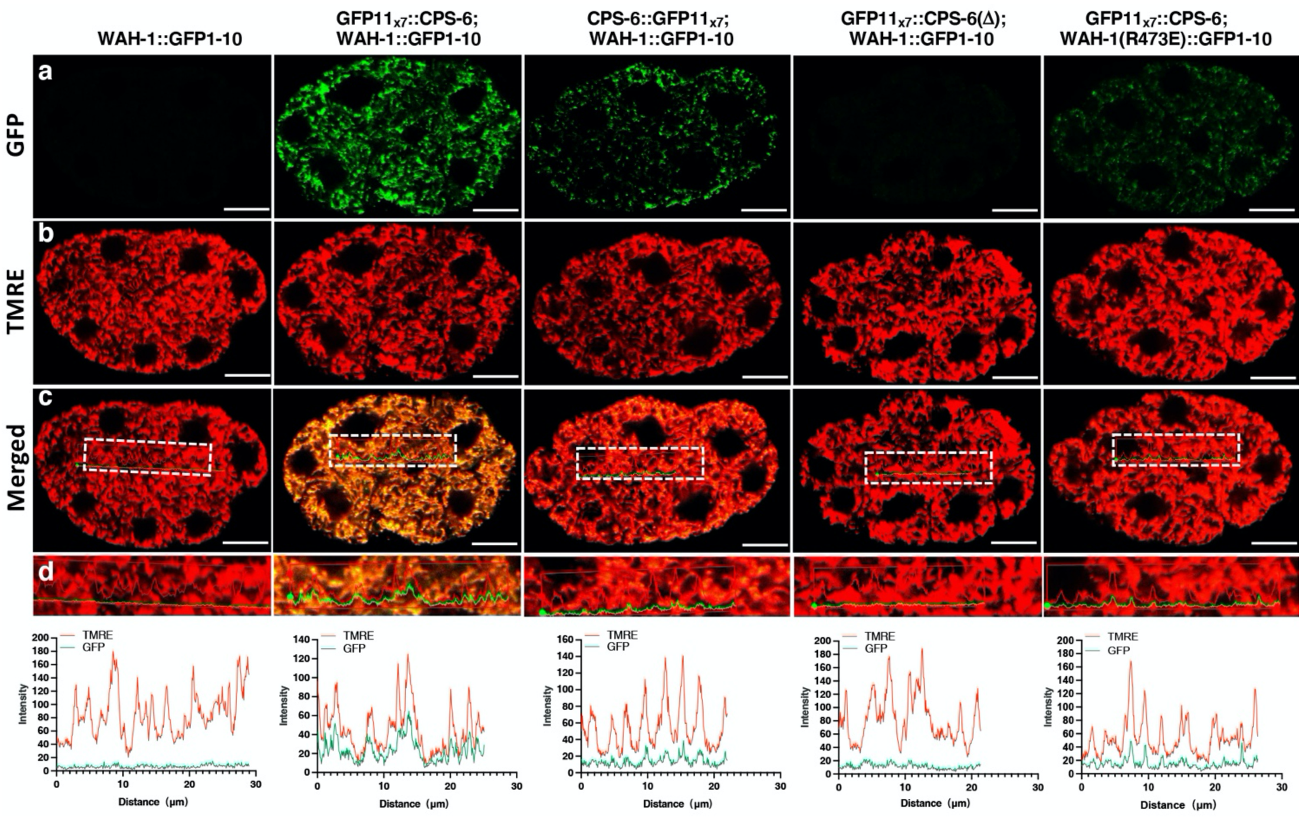

Fig. S2. WAH-1 and CPS-6 interact in mitochondria.

a-c Representative GFP, TMRE, and merged images of embryos carrying the indicated knockins at the stage of 6 to 8 cells. Exposure time, laser strength, and other settings were identical in all embryos. Scale bar represents 10 μm.

d Enlarged images of the areas highlighted in dashed boxes in **c**, with the fluorescence intensity peaks of TMRE and GFP across the area shown below.

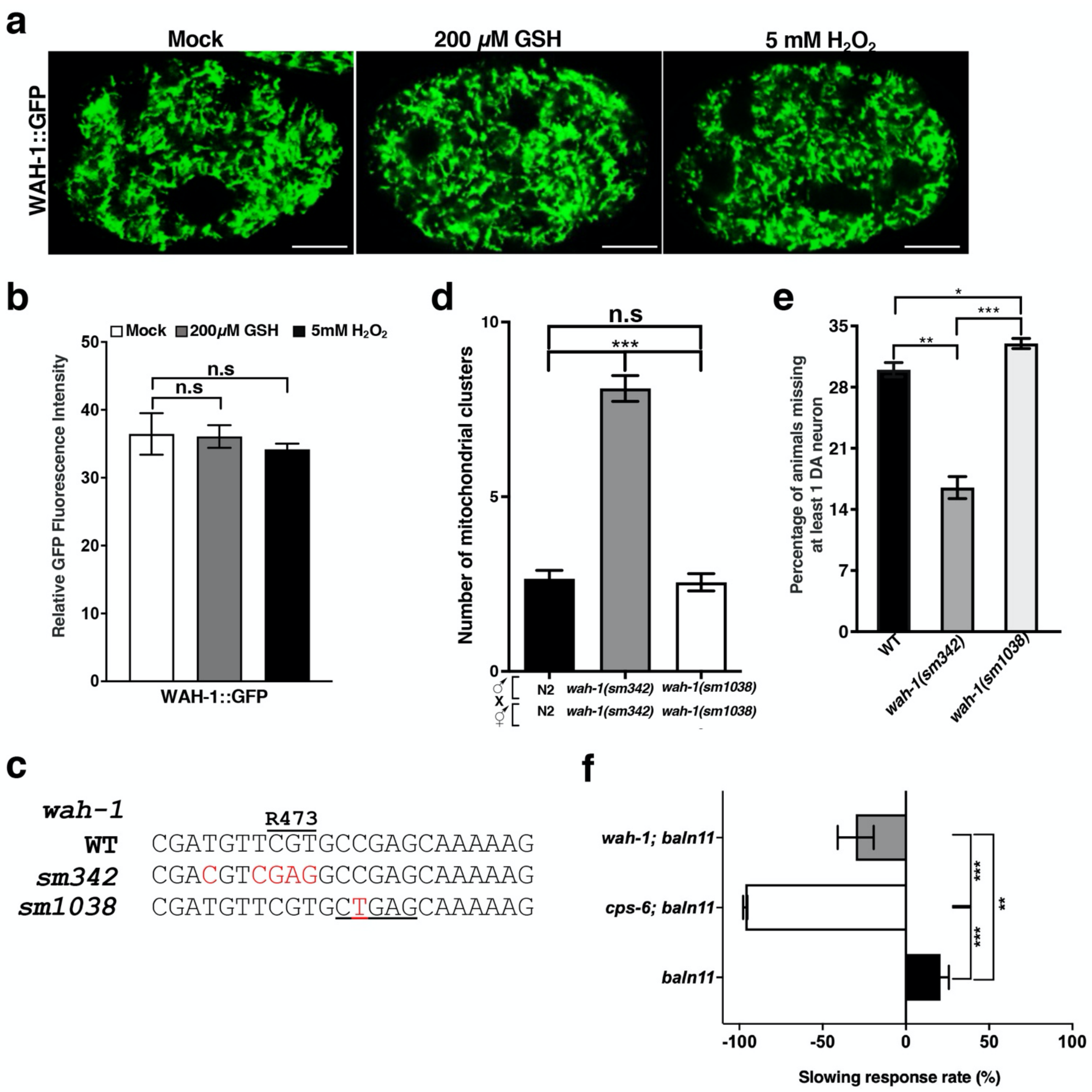

Fig. S3. WAH-1::GFP expression under different redox conditions, the *wah-1* revertant, and the enhanced slowing response locomotion assay.

a Representative WAH-1::GFP fluorescence images of embryos with the indicated GSH and H_2O_2 treatment. Exposure time, laser strength, and other settings were identical for all embryos. Scale bar represents 10 μm. **b** Quantification of relative GFP fluorescence intensity of embryos as shown in **a**, n =10 embryos for each condition. **c** Alignment of the *wah-1* DNA sequences in N2 (WT), *wah-1(sm342[R473E])*, and *wah-1(sm342 sm1038[E473R])* animals. Nucleotides altered (in red) in two *wah-1* alleles and the Arg473 codon are shown. The sequence underlined indicates creation of a Dde I restriction enzyme site. **d** Quantification of MTR-stained paternal mitochondrial clusters in

64-cell stage embryos from the indicated cross of MTR-stained males with unstained young adult hermaphrodites. Data are means ± SEM; n = 20. **e** Comparison of DA neuronal loss in adult day 2 *baIn11* animals in the indicated genetic background. Data are mean ± SEM, n = 50 animals per experiment. Four independent experiments were performed for each genotype. **f** Enhanced slowing response rate of adult day 2 *baIn11* animals in the indicated genetic background (see Materials and methods for detail). Data are mean ± SEM, n=20-30 animals per experiment. Six independent experiments were performed for each genotype. Statistical significance was determined using One-way ANOVA test, *$P < 0.05$, **$P < 0.01$ *** $P < 0.001$. n.s, not significant (**b, d-f**).

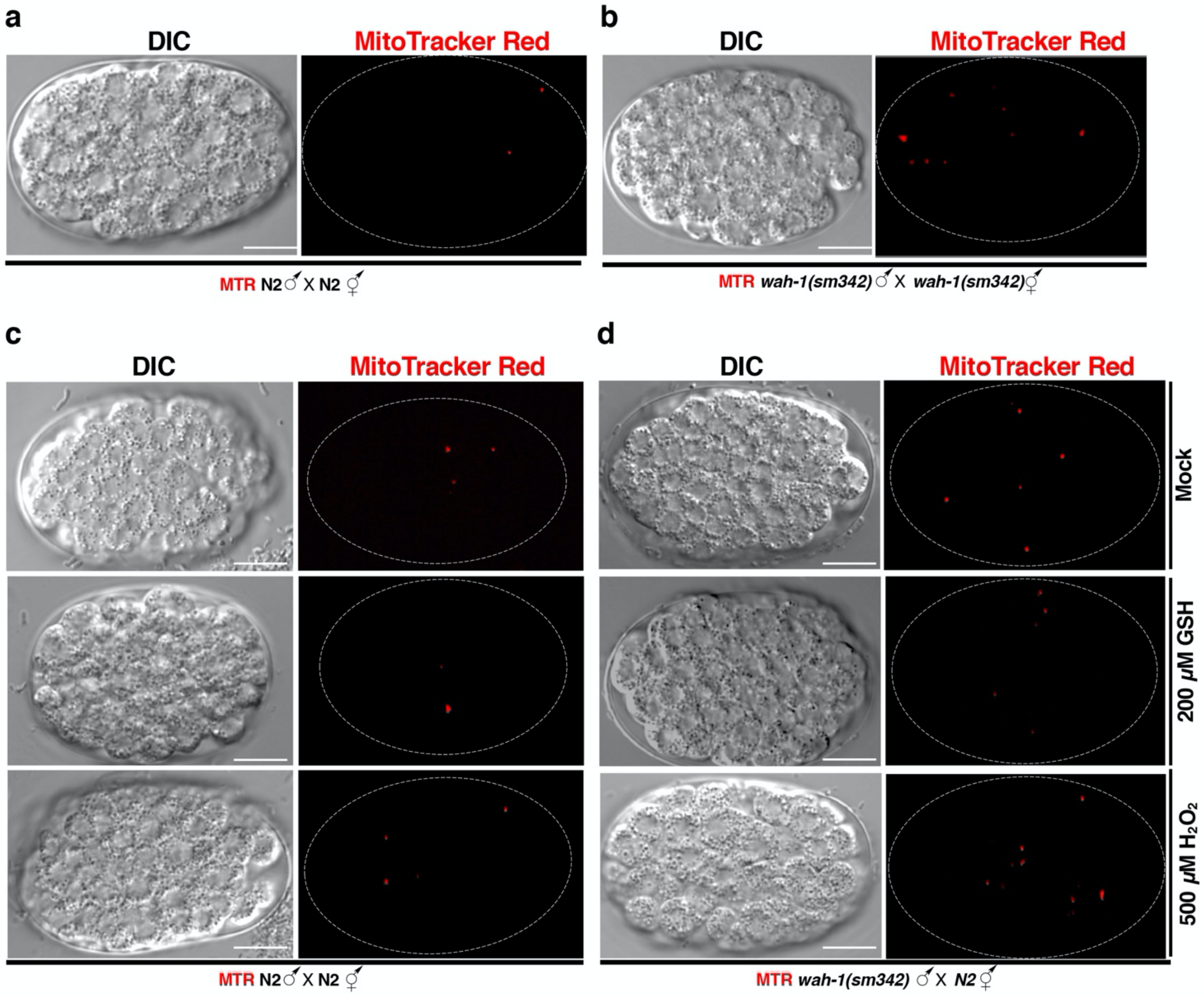

Fig. S4. ***wah-1(sm342[R473E])*** **delays PME in** ***C. elegans*****. a-d** MTR-stained paternal mitochondrial clusters in 64-cell embryos from the indicated crosses with MTR-stained males without or with the indicated treatment. Cross-fertilized embryos dissected from mated hermaphrodites were imaged. Representative differential interference contrast (DIC) and MTR staining images of the embryos are shown. The red dots are paternal mitochondrial clusters stained by MTR. Scale bars represent 10 μm.

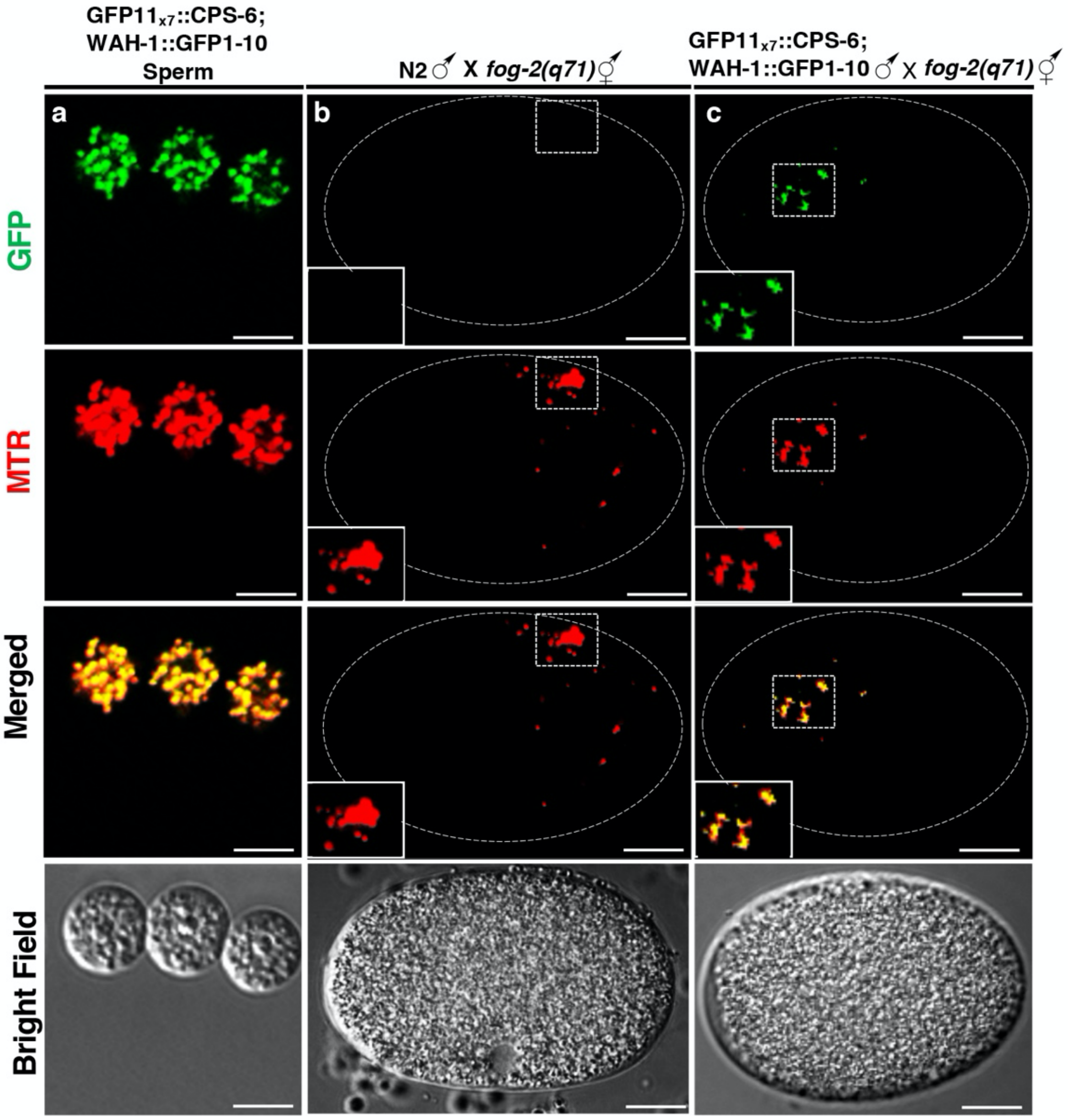

Fig. S5. WAH-1 and CPS-6 maintain interaction in mitochondria after fertilization.

a-c Representative GFP, MitoTracker Red (MTR), GFP/MTR merged, and bright-field images of spermatozoa released from the gonad of the indicated male prestained by MTR (**a**) and zygotes from the indicated crosses between MTR-prestained males and unstained fog-2(*q71*) females (**b, c**). Enlarged images of the regions highlighted by dashed boxes are shown at the left bottom. Scale bars represent 10 μm.

a. Template sequences to generate *gfp11*$_{x7}$*::cps-6* and *gfp11*$_{x7}$*::cps-6(△)* knockins

atccaagaattgtgatggatctgcaggccttacgtgcacatttcccggtgattgccgaatcggagacactgtaaaagtgaattgt
acttcacggaaaggttgcccgaatcctgtcagcagaaataatgtcgaagcggtgtgtcggttttgttggcaacttctacctgggg
attatgattgcgagccagcgacaaattgctccacgagtagtacaaagcttctcgtgacaaagtgctcggtgagcaatttctttaa
tttaatatctgttttaaaatacctaattttcaaaaaaaaattgaattttcaggctcacagctcagtaatatgtatgggacaacgtaatt
tctacaagcgaattccatgtaactggtcatctggatacagttggacaaaaacaatgattctctcagttgtgctcggcggttttgga
gctgatcgtttctacttgggactctggaaatctgcaattggaaaacttttcagtttcggtggtcttggtgtgtggactcttgtggacgtt
gttctgatcgcagttggatacattaaaccttacgatggatccatgtatatttgattttttttttaattttcttttccaatttgtgtaaatacttc
ctgtaataagcttaagcttcgggtgatttctgatactcttgttattctccttattttcgtctttttgaaattgccatttttccattaaaagtaat
aaatatttctacaaaattcgattgtaaactgtttgaatatgaagtagattattactcaaaaatatctttttttacatgaattttcagtttttct
atttttcttttccaaattttccaattttaatttccagccgaaatATGATCGGCAAGGTGGCTGGAACCGCGGCA
ATCGCTGGAATATCTTTCCTCGCTGGAAAATATTCGAATGACGATCTGCCAATCTTCAG
GAATGTTCAATCAGCCACCAATGTTCCAATGgtttaataaaaatcttctagaatcctgaaaaatgccgctttt
gcagAATCAAATCCAAGTTTCAGAGCCAATGACA**CGTGACCACATGGTCCTTCATGAGT**
ATGTAAATGCTGCTGGGATTACAGGTGGCTCTGGAGGTAGAGATCATATGGTTCTCC
ACGAATACGTTAACGCCGCAGGCATCACTGGCGGTAGTGGAGGACGCGACCATATG
GTACTACATGAATATGTCAATGCAGCCGGAATAACCGGAGGGTCCGGAGGCCGGGA
TCACATGGTGCTGCATGAGTATGTGAACGCGGCGGGTATAACTGGTGGGTCGGGCG
GACGAGACCATATGGTGCTTCACGAATACGTAAACGCAGCTGGCATTACTGGCGGA
TCAGGTGGCAGGGATCACATGGTACTCCATGAGTACGTGAACGCTGCTGGAATCAC
AGGCGGTAGCGGCGGTCGGGACCATATGGTCCTGCACGAATATGTCAATGCTGCCG
GTATCACCGGCGGCGTAAAACCAGCATCTCTAAATGCTGATGCAATGGGACCATCTCG
TTCGGCAGAGATCATGAAGCACGGATATCCCGGATTCACAAATGTTCGAACTTATGAG
GATTTCGTGCTTTCCTACGATTATAAGACAAGAACTGCACACTGGgttagtagaaaattatgaga
aaatatatttcaaaaatctataccggacgccaagttttagcgccaaaaatacggtacccagcagggctactgcaaaaataaa
tcttccaccaaaaaaaaatttgtttcatttttgctgaatcagtgttgtaatttatttttggtttttgtcccgaaaaaccaaaaattttttt
ttccaaaaacaaaggcagctttggtctcgacatgccttttgtttttgtataaaacaaatgggtgtgcgcctttaaaaagtactgtcat
ttcaaactatcataatttttatttttagtattttattttaaaaaatatttatttatttaaaaaaacacattaattttaacaatcgtgaaaaaa
aactataaaaatcaatgaatattccacaacaacgaaactttgaacctaccgtactatttaaaggtgcacacctttctacattaaa
aaattgtcgtgtcgagaccggttttaataaaaaattgaatttccagGTCTGCGAACATCTTACACCAGAACGA
TTAAAACACGCAGAAGGTGTGGATCGGAAGCTTTGTGAATTCAAGCCAGATATCACTT
TCCCGCAGAAATTCCTTTCTCAAAATACGGATTATAAGgttagttcttcgtaattattacataaaaaatatt
tctgaaccatccaatgtttcagTGCAGTGGATTCGATCGTGGACATCTCGCCGCCGCTGGAAATC
ATCGGAAGAGTCAGTTGGCGGTCGATCAAACTTTTTATTTGAGCAATATGAGCCCACA
AGTTGGACGAGGATTCAATCGCGATAAGTGGAATGATTTGGAAATGCACTGCAGgttggt
aaaataaataaagtttttttgccgggaaaaaaaacgcgaaaaatgtgaaataatttttttttctcgtcgacgaagtttttcgcaaaaac
acgtaactctaaagaattttgtttttttgaaaaattaatacaatttgaaacttttggaaaatttttttgagaaaaaaaaaacgaaaaatc
tacagcccagtctgaaaccagctggtaccaagatttttctttacaatactaaaaataattttctacaatttccagAAGAGTCG
CAAAAAAGATGATCAACTCCTACATAATCACCGGTCCCCTCTACCTTCCAAAACTTGAA
GGAGATGGAAAAAAGTATATCAAGTATCAAGTTATCGGTGATAATAACGTTGCAGTGCC
AACTCATTTCTTCAAAGTTGCTCTTTTCGAAGTGACACCCGGCAAATTCGAGCTGGAA
TCCTATATTTTGCCAAACGCAGTCATCGAGGATACTGTAGAAATTTCCAAGTTCCATGTT
CCATTGGATGCTGTGGAGAGAAGTGCGGGTCTTGAGATTTTCGCTCGTTTGGACCCT

AAGAGCATTGTGAAAGAGAACGGAGCAAAGAAGGGAGGATTACTTTGGTGAttgattggatt gtgattccggtgaattttaggtttaggttgttttgtttttgtttcacaaataaatttttgttaactttttgatttactgattgaaaaaacttga aaactcaattaaaaagaacaataccaatagaaattttaaaatcttaacttact

b. Template sequence to generate *cps-6::gfp11*$_{x7}$ knockin

TTCGATCGTGGACATCTCGCCGCCGCTGGAAATCATCGGAAGAGTCAGTTGGCGGTC GATCAAACTTTTTATTTGAGCAATATGAGCCCACAAGTTGGACGAGGATTCAATCGCGA TAAGTGGAATGATTTGGAAATGCACTGCAGgttggtaaaataaataaagtttttttgccgggaaaaaaacgc gaaaaatgtgaaataatttttttctcgtcgacgaagtttttcgcaaaaacacgtaactctaaagaattttgtttttgaaaaattaat acaatttgaaacttttggaaaattttttgagaaaaaaaaacgaaaaatctacagcccagtctgaaaccagctggtaccaaga tttttctttacaatactaaaaataattttctacaatttccagAAGAGTCGCAAAAAAGATGATCAACTCCTACAT AATCACCGGTCCCCTCTACCTTCCAAAACTTGAAGGAGATGGAAAAAAGTATATCAAGT ATCAAGTTATCGGTGATAATAACGTTGCAGTGCCAACTCATTTCTTCAAAGTTGCTCTTT TCGAAGTGACACCCGGCAAATTCGAGCTGGAATCCTATATTTTGCCAAACGCAGTCAT CGAGGATACTGTAGAAATTTCCAAGTTCCATGTTCCATTGGATGCTGTGGAGAGAAGT GCGGGTCTTGAGATTTTCGCTCGTTTGGACCCTAAGAGCATTGTGAAAGAAAACGGA GCAAAGAAGGGAGGATTACTCTGG**GGTGGACCAGGAGGTGGTGGAAGTCGTGACC ACATGGTCCTTCATGAGTATGTAAATGCTGCTGGGATTACAGGTGGCTCTGGAGGTA GAGATCATATGGTTCTCCACGAATACGTTAACGCCGCAGGCATCACTGGCGGTAGTG GAGGACGCGACCATATGGTACTACATGAATATGTCAATGCAGCCGGAATAACCGGA GGGTCCGGAGGCCGGGATCACATGGTGCTGCATGAGTATGTGAACGCGGCGGGTA TAACTGGTGGGTCGGGCGGACGAGACCATATGGTGCTTCACGAATACGTAAACGCA GCTGGCATTACTGGCGGATCAGGTGGCAGGGATCACATGGTACTCCATGAGTACGT GAACGCTGCTGGAATCACAGGCGGTAGCGGCGGTCGGGACCATATGGTCCTGCAC GAATATGTCAATGCTGCCGGTATCACCGGCGGC**TGAttgattggattgtgattccggtgaattttaggttt aggttgttttgtttttgtttcacaaataaatttttgttaactttttgatttactgattgaaaaaacttgaaaactcaattaaaaagaaca ataccaatagaaattttaaaatcttaacttactattagtcaacaacaaaaattcatccgaaataggggaaggtggaaaaggat accagaaaggaatttcatacggcaaaaaaatttggatgaaaaacaatatccctatattgaaagtgagacattattttatgtcact agttttatgtaatatgaaattcctttcaccgagagaaaaatattttagaaaagaaaaagtaaattaaagggaacagggaggaa acaaaaacacgagtatatttgggaatcttaacgagtcgatgagacagagctcaaagttctgtcttttgttccggcgactttctcct cagcaacagcttcagcgatgaacattttcgtgatattttcgctggtcacagtcttcgagaagtcagcgagagcgttggtgtcaaa actggcgagaagctcatccaaaagtccatttcttgtttggagttg

c. Template sequence to generate *wah-1::gfp1-10* knockin

gtactcaagaaagctaaatttatcaaaaaaaaatctatacatgttctgaaaaaatgacaatttaccaagtaattttgcaagacc atttcttagGTACCAACCATCATTCTTCACCAAATTCGCCCCACATCTGCATATCAACGCGAT CGGAAAATGCGATTCCTCACTGGAAACCGTATCAGTTCATGCTGAACCAGACAAGGAT ACGCCTCTTGAGAAGGCTGTCGTATTTTATAAATCGAAAGAAGATGGAAGTgtaagttgtga caagcctgaaaaattattccaaaatgtccaaaaatgtcgaaaaaggtgaaaaaaaatgtccaaaaatgtccaaaaatgtag aaaaacgtctaaaaatgtctaaaaatgtccaaaaatgtagaaaaacatctaaaaaagtctaaaaatgccgaaaaatgtag aaaaaagtctaaaaatatagttaaattttaaaaaatgctgaaaaatgccaagaaaaatctctgaaaatgtagaaaatgtctaa aatttaaggaaaatgtacgaaaatgccaaaaaaaatgtccacaaatgccttaaaatgtaggaaaatgtttgaaaaatgccaa

aaaaaatgtcgaaaaatggctaaaaatgtctaaaaatatagattaatgtctaaaaatgtaggaaaacgtaggaaaatgtcta
aaaatgtccaaaaatgtcgaaagatgcctaaaaaatgtctaaaaatgcagaaaaatgtagaaaaatgtagaaaatggtcc
aaaaatgtctaaaaatttagaaaagggccaaaaaatttagaaaaaagtccaaaagtgaaggaaaatgtttaaaaatgttaa
aaaatgtcggaaaatgtagataaatgtccgaaaatttcgaaaaaagtctgaaaatgtcaaaaaaaaaattcaaaaaaaaa
gtaggaaaatgccaaaaattcagaaaaatgtagaaaaatcccataataaataattttccagATCGTCGGTGTCCTA
CTCCTGAACGTGTTCGGACCTTCACTGGATGTAGCTCGGCGTATAATTGATGATAGGA
AGAAAGTGGATGAGTACAAGGAAATTGCAAAGCTTTTCCCACTTTATGATCCAGTAAAA
AGTGATGAAGACGATGCGAAGAGTGCT**GGTGGACCAGGAGGTGGTGGAAGTATGTC**
CAAAGGAGAAGAACTGTTTACCGGTGTTGTGCCAATTTTGGTTGAACTCGATGGTGA
TGTCAACGGACATAAGTTCTCAGTGAGAGGCGAAGGAGAAGGTGACGCCACCATT
GGAAAATTGACTCTTAAATTCATCTGTACTACTGGTAAACTTCCTGTACCATGGCCGA
CTCTCGTAACAACGCTTACGTACGGAGTTCAGTGCTTTTCGAGATACCCAGACCATA
TGAAAAGACATGACTTTTTTAAGTCGGCTATGCCTGAAGGTTACGTGCAAGAAAGAA
CAATTTCGTTCAAAGATGATGGAAAATATAAAACTAGAGCAGTTGTTAAATTTGAAGG
AGATACTTTGGTTAACCGCATTGAACTGAAAGGAACAGATTTTAAAGAAGATGGTAA
TATTCTTGGACACAAACTCGAATACAATTTTAATAGTCATAACGTATACATCACTGCTG
ATAAGCAAAAGAACGGAATTAAAGCGAATTTCACAGTACGCCATAATGTAGAAGATG
GCAGTGTTCAACTTGCCGACCATTACCAACAAAACACCCCTATTGGAGACGGTCCG
GTACTTCTTCCTGATAATCACTACCTCTCAACACAAACAGTCCTGAGCAAAGATCCA
AATGAAAAATAGagatttagatttttagggttaatatatttaattttttttatttttttatttttgttgttttttttggttttttctcagctgttt
tcccccgaatttcatgtttgtttggaccccatgacaataaaaactgtattttttaaaccaattaatttattcacaacttaacagacaa
caaatgccgagtggcaagaagctccatacaagtatggctatcctcaatcgacgaatgacccgacgcttccgactgaacatc
cttacacagatatttcttcgcaagattctgaagggacaacttgagaatgtaggatctggatggatcgacgatcgagaacagga
ctgcagtgtcgatgacattgtggtggacaaggcggagagctttcagatccgattccagggagtgaccgacgagcagggtttc
agagttgatgagttggaagagctgcaggcgacactgcaaattttggggttttaggattttagagcaattgggattttttttcagatc
aggggtaggcggctagcgatttttccggcaatcggcaaaatggcggaattatacatttctggcaaatcggagaattgtcggaa
ttgaaaatttccggcaaatcgacaattacgcaaattgccggaattgaaatttctggcaaatcggcaaactgccggaattgaaat
ttcgggcaaaccggcaaactgccggaattatacatttctggcaaatcggagaattgccggaattgaaaatttccggcaaatcg
gtaaataagcaaattgccggaattgaaattccggcaaatcggcaaattgccggaattgaacattttcggcaaatcggtaatcc
ggcaagttgtttttaatgagttggaaaatcattgaaaattccgcagaaattacagtgctcttta

d. The sequence altered in *wah-1(sm1035[R473E]sm984[wah-1::gfp1-10])* knockin

WT   CGATGTTCGTGCCGAGCAAAAAGTGGAAGGAGTCCGGAAAT
R473E   CGATGTT**GAA**GCTGAGCAAAAAGTGGAAGGAGTCCGGAAAT
R473E

Fig. S6. Nucleotide sequences of the templates used for generating knockins.
a-d The uppercase letters show the exon sequences of the *cps-6* and *wah-1* genes and the lowercase letters indicate the intron or untranslated sequences. Synonymous mutations are marked in red. Sequences encoding *gfp11*$_{x7}$ and *gfp1-10* are highlighted in green. Sequences highlighted in purple indicate flexible linkers. The sequence underlined (**a**) indicates the region of the *cps-6* gene deleted in the *gfp11*$_{x7}$*::cps-6(*Δ*)* knockin. Alignment of the sequences containing nucleotides altered (in

bold) in the *wah-1(sm1035[R473E]sm984[wah-1::gfp1-10])* knockins is shown (**d**), in which WT is the sequence from N2 animals and the sequence underlined indicates creation of a Dde I restriction enzyme site.

Table S1. sgRNA sequences used to generate knockins

Knockin	sgRNA	sgRNA sequence
cps-6(sm822[gfp11$_{x7}$::cps-6])	sgRNA 1	GCAGAATCAAATCCAAGTTTCGG
	sgRNA 2	CCGTAAAACCAGCATCTCTAAAT
cps-6(sm995[gfp11$_{x7}$::cps-6(Δ)])	sgRNA 1	CCAGAACGATTAAAACACGCAGA
	sgRNA 2	GAAAGAGAACGGAGCAAAGAAGG
cps-6(sm1033[cps-6::gfp11$_{x7}$)	sgRNA 1	CAAAGAAGGGAGGATTACTTTGG
	sgRNA 2	TGATTGATTGGATTGTGATTCCGG
	sgRNA 3	AAGAGCATTGTGAAAGAGAACGG
wah-1(sm984[wah-1::gfp1-10])	sgRNA 1	CCAGTGAAAAGTGATGAAGACGA
	sgRNA 2	GCTTAGAGATTTAGATTTTTAGG
wah-1(sm342[R473E])<br>*wah-1(sm342 sm1038[E473R])*<br>*wah-1(sm1035[R473E]sm984[wah-1::gfp1-10])*	sgRNA 1	ATTGAAGCAATCAGGAAGGGTGG
	sgRNA 2	CCGAGCAAAAAGTGGAAGGAGTC

The sgRNA sequences targeting regions of the *cps-6* gene or the *wah-1* gene are shown.

Table S2. PCR primers used to screen for the knockins

Knockin name	forward PCR primer	reverse PCR primer
cps-6(sm822[gfp11$_{x7}$::cps-6])	ATCCAAGAATTGTGATGGATC	CTTGTGGGCTCATATTGCTC
cps-6(sm995[gfp11$_{x7}$::cps-6(Δ)])	CAGCTTTGGTCTCGACATGCC	CAACTCCAAACAAGAAATGG
cps-6(sm1033[cps-6::gfp11$_{x7}$)	TCGATCGTGGACATCTCGCC	ACTCCAAACAAGAAATGGAC
wah-1(sm984[wah-1::gfp1-10])	CTCAAGAAAGCTAAATTTATC	GAGCACTGTAATTTCTGCGG
wah-1(sm342[R473E])<br>*wah-1(sm342 sm1038[E473R])*<br>*wah-1(sm1035[R473E]sm984 [wah-1::gfp1-10])*	CCACCGACCAATCAGCGATTC	GCTTTTCCCTCCGACTTCCC

The PCR primers used to screen for the *cps-6* or the *wah-1* knockin are listed.
